# Mucous membrane pemphigoid in HIV patient: a case report

**DOI:** 10.1186/1757-1626-1-345

**Published:** 2008-11-22

**Authors:** Adriana Demathé, Lívia T Arede, Glauco I Miyahara

**Affiliations:** 1 Department of Pathology and Propaedeutics, School of Dentistry, Araçatuba Campus, São Paulo State University, Araçatuba, São Paulo, Brazil; 2 Professor Department of Pathology and Propaedeutics, School of Dentistry, Araçatuba Campus, São Paulo State University, Araçatuba, São Paulo, Brazil

## Abstract

**Background:**

Mucous Membrane Pemphigoid (MMP) is a rare group of chronic autoimmune disorders characterized by blister producing lesions. It is the variant most likely to occur in the oral cavity and eyes. Few studies shows autoimmune bullous diseases associated with human immunodeficiency virus (HIV).

**Case presentation:**

oral blisters, painful ulcerations and eye symblepharon were observed in a patient with HIV infection. The histopathologic exam showed a subepidermal blister and the overall features were suggestive of MMP.

**Conclusion:**

In this case, the MMP was associated with HIV. Interactions with the immune system may lead to the development of this autoimmune process.

## Background

Mucous membrane pemphigoid (MMP), new denomination of cicatricial pemphigoid [1,2] encompasses a group of chronic subepithelial autoimmune blistering diseases that predominantly affect the mucous membranes with potential scar formation. This is a rare condition and few studies shows autoimmune bullous diseases associated with human immunodeficiency virus (HIV). We report the case of a patient with HIV infection who developed mucous membrane pemphigoid in the absence of any other HIV-related clinical conditions that is, to our knowledge, the first description in the medical literature.

## Case presentation and discussion

A 48-year-old man diagnosed with HIV infection 13 years earlier, was presented with tense oral blisters and painful ulcerations associated with 15 days' duration. He had no history of skin or genital erosions. The patient showed no symptoms of HIV-related illnesses and never used antiretroviral therapy. Your last CD4 count was 1970/mL.

**Figure 1:**
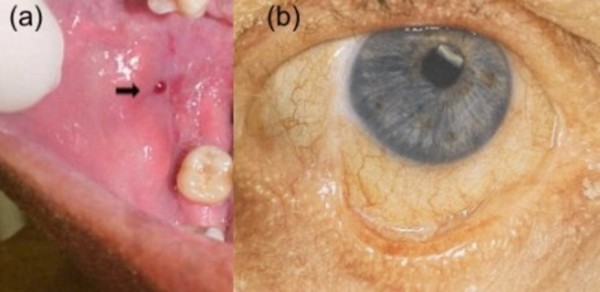
(a) A man with HIV presenting oral blister and erosions; (b) symblepharon formation on eye.

The patient was not taking any other prescription or alternative medicines. During the past 2 years, he experienced 7 episodes of intense pain and burn in buccal mucosa, including the present episode of bullous lesions. On oral examination, he had few well-defined erosions involving mouth and a small blister (Fig 1a). In his left eye its possible to see a mild conjunctivitis and symblepharon formation (Fig 1b). Nikolsky sign on buccal mucosa were positive.

**Figure 2:**
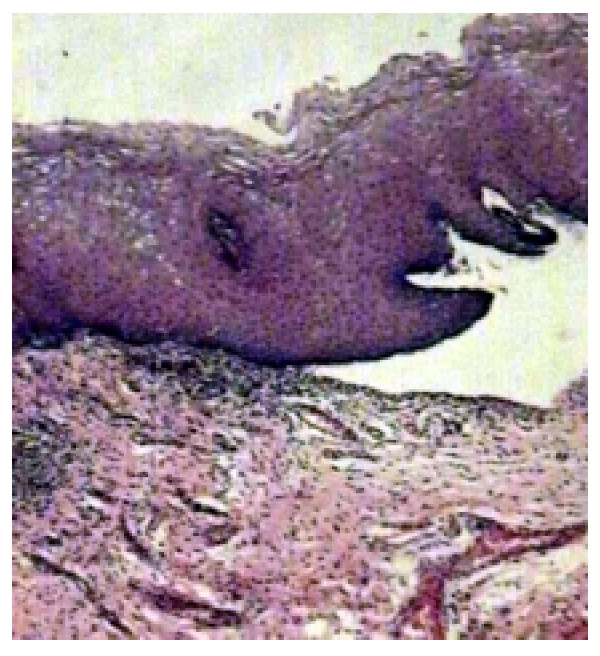
**Subepidermal bullae**. Dermis shows perivascular inflammatory infiltrates containing eosinophils and neutrophils (HE, original magnification ×100).

The histopathologic exam of perilesional tissue showed on a subepidermal blister with an inflammatory infiltrate in the upper dermis composed of lymphocytes and eosinophilic leucocytes (Fig 2).

The overall features were suggestive of mucous membrane pemphigoid. In the absence of immunofluorescence studies, in this case, the diagnosis was firmly based on a classic clinical presentation of MMP: severe vesicles, erosions and crusts on mucous membranes, typically affecting the oral cavity and eyes [3]. A direct immunofluorescence test could not be done because the patient was a prisoner and the pathology laboratory of the prison camp does not have the necessary technology for immunofluorescence studies.

The possibility of a drug-induced bullous eruption was effectively excluded in this patient because did not use any drugs. In this prison exists a specific set for patients in medical observation. In this local, the patient stay isolated alone, and he is reviewed before entering in this place, so that the specified treatment can be appropriately made.

He was treated with topical corticosteroids and informed about oral hygiene care. Within 2 weeks of treatment, he had significantly improved with almost complete disappearance of the lesions. A few small, tense vesicles continued to appear eventually.

Oral corticosteroid therapy in HIV-infected individuals raises concern regarding increased immunosuppression, therefore it should not be used in continuous treatment.

Autoimmune bullous diseases in oral cavity associated with human immunodeficiency virus (HIV) infection are extremely rare and illustrates that patients with HIV are capable of mounting organ-specific autoantibody-mediated diseases [4-7].

Molecular mimicry is one of the proposed mechanisms in the development of autoimmune disease in HIV patients. The exogenous infectious agent may have molecular similarity to a self-antigen and may therefore induce an autoimmune response [8].

Immune deregulation secondary to HIV infection may lead to increased risk of developing an autoimmune disease. Development of autoantibodies may be part of the nonspecific polyclonal B-cell stimulation seen in early stages of HIV infection secondary to interleukin (IL)-1 and IL-2 release by HIV-infected macrophages [9].

On the other hand, the loss of specific immunomodulatory CD4 T cells may allow the expansion of the B-cell clone responsible for the autoantibody formation [7].

Immunodeficiency and autoimmunity seem to be the two opposite sides of the spectrum of the clinical immune response. However, persistent antigen stimulation, due to an inherently defective immune system ability to eradicate pathogenesis is the primary cause leading to autoimmunity in patients with immunodeficiency states [10].

## Conclusion

This case and others of autoimmune bullous diseases in association with HIV illustrated on literature showed that HIV might have varying interactions with the immune system, which may lead to the development of autoimmune processes.

## Consent

Written informed consent was obtained from the patient for publication of this case report and accompanying images. A copy of the written consent is available for review by the Editor-in-Chief of this journal.

## Competing interests

The authors declare that they have no competing interests.

## Authors' contributions

AD and GIM analyzed and interpreted the patient data regarding the autoimmune bullous disease and the HIV infection. LTA was a contributor in writing the manuscript. All authors read and approved the final manuscript.
